# Irish pig farmer’s perceptions and experiences of tail and ear biting

**DOI:** 10.1186/s40813-019-0135-8

**Published:** 2019-12-17

**Authors:** Amy Haigh, Keelin O’Driscoll

**Affiliations:** 1 Present address: School of Biology and Environmental Sciences, University College Dublin, Dublin, Ireland; 2 Pig Development Department, Centre for Grassland Research and Innovation, Teagasc, Fermoy, Co. Cork Ireland

**Keywords:** Condemnation, Welfare, Enrichment, Farmer attitudes, Survey, Abnormal behaviour

## Abstract

Abnormal behaviours such as ear and tail biting of pigs is of significant welfare and economic concern. Currently, pig welfare legislation is under renewed focus by the EU commission and is likely to be enforced more thoroughly. The legislation prohibits routine tail docking and requires adequate enrichment to be provided. In Ireland, tail-docking is still the most utilised control mechanism to combat tail biting, but biting is still widespread even in tail-docked pigs. In addition, as pig farms are almost all fully slatted, bedding type material cannot be provided. Thus, the opinions, and practices of farmers in countries like Ireland, which may need to make significant adaptations to typical pig management systems soon, need to be considered and addressed. We carried out a survey of pig farmers during 2015 in order to gain a greater understanding of the extent of biting on Irish farms, perception on the most important preventive measures, current enrichment use and actions following outbreaks. Fifty-eight farmers from 21 Counties responded with an average herd size of 710 ± 597 sows (range 90–3000 sows). Only two farms had experienced no biting in the last year. Of the farms that had experienced tail biting (88%), 86% had also experienced ear biting. The most common concerns relating to biting were condemnation and reduced productivity of bitten pigs with both receiving an average score of 4 (most serious). Ear biting occurred most commonly in the 2nd stage (approximately 47–81 days from weaning) weaner and tail biting in the finishing stage. The most important preventive measures were felt to be taking care of animal health, restricting density, maintaining an even quality of feed/content and maintaining good air movement. Sixty-five percent of respondents added additional enrichment following an outbreak. Chains were the most common form of enrichment currently used (83%). Those not using chains favoured wood, toys and rope (17%). Identification of the most effective and accessible control and prevention measures both for the animals and for the farming community is thus essential. Improved understanding of the concerns and practices of producers, which this survey contributes to, is a first step towards this aim.

## Background

Despite widespread tail docking (>99% of pigs) evidence of tail biting is prevalent in Irish pig production, with tail lesions observed in 58.1 and 72.5% of pigs at the abattoirs [1, 2]. More recently, moderate and severe (complete removal of the tail) lesions were observed in 25.2 and 3.1% of Irish abattoir pigs [3]. The proportion of Irish pigs with ‘moderate’ damage is like the 34.6% of Finnish pigs observed to have tail damage [4], even though Finnish pigs are undocked. Likewise, Keeling et al. [5] also recorded low levels of damage in undocked pigs in Sweden; the overall prevalence of injury or shortening of the tail was 7.0 and 7.2%, respectively, and for severe injury (≤ half of the tail left), these percentages were 1.5–1.9%. In both Finland and Sweden tail docking is forbidden, and thus the lower levels of tail injury are likely due to better management than in Irish pig farms. For instance, in Sweden there is a ban on fully slatted floors in all pig housing, as well as higher standards for air quality and light provision than the EU standard [6].

Tail and ear biting lesions have a negative effect on both animal welfare and the economics of the pig farm [7]. Tail biting injuries can be minor, or so severe that the tail is bitten to the rump and the animal may need to be euthanized [8]. The average daily weight gain of bitten pigs is lower than unbitten (1–11% lower; [9–12]), as is carcass weight [2] Victims are also likely to suffer more frequently from other health disorders than pigs with non-bitten tails (leg disorders and arthritis, [10, 13]).

Not only are substantial losses encountered due to carcass weight reduction, but also due to carcass condemnation [14]. Secondary infection may occur in the lungs, and less commonly in the kidneys and other parts of the body, as a result of pyaemia [15]. Furthermore, wounds caused by tail biting can lead to an increased risk of infection and have been associated with being important in the primary transmission of trichinosis [15].

Tail biting is also frustrating for producers as its main risk factors vary between herds making it difficult to pinpoint the best preventive measures, as it is believed to have multifactorial causes [16]. Factors such as stress [17], restricted feed access [18–21], insufficient diet [15], high stocking density [16, 22, 23], early weaning [15], poor ventilation/incorrect temperature [24], breed [11, 25, 26] and lack of enrichment [27–32] are all hypothesised to play a part in causing biting outbreaks. Indeed 25 different hazards were used in the EFSA [33] risk assessment for tail biting, and 83 risk factors were identified by Taylor et al. [34] using a combination of literature review and expert opinion.

Although there is not as much research on the aetiology of ear biting as tail biting, it has been suggested that both are linked [9]. Brunberg et al. [35] observed that tail biting pigs performed a higher frequency of ear biting than non-performers. Bracke et al. [24] carried out a survey of Dutch producers and found that biting of both tails and ears were identified by the farmers as a welfare problem in pig farming. Van Putten [36] argues that ears and tails are the easiest to chew, but ear-chewing is more likely to provoke an attack by the recipient. Therefore, incidences of ear biting may be lower than that of tail biting or occur at different stages.

Valros et al. [20] emphasised the importance of listening to farmers in order to fully understand the problems of tail biting and to enhance communication between science and end users. While in Ireland factory data has confirmed that biting and specifically tail biting does occur [2, 3], less is known about the action undertaken by Irish producers to treat tail biting, their biggest concerns and their experience of what the best preventive measures to outbreaks on their farm are. Currently, there are strong moves by the EU commission to encourage enforcement of Directive 2008/120/EC, which stipulates that routine docking of pigs should not be carried out, and pigs should be provided with appropriate environmental enrichment [37]. In order to gain a greater understanding of the extent of biting on Irish pig farms, the perceived best preventive measures to biting outbreaks and current enrichment practises and attitudes to different forms of enrichment, we conducted a survey amongst Irish producers in 2015.

## Methods

A survey was developed through discussions with pig advisors, researchers and technicians in the Teagasc Pig Department, and based upon the survey of Valros et al. [20]. An initial draft was distributed to producers at the 2015 Pig Department Research Dissemination Day, and questions which proved difficult to interpret amended prior to the main survey taking place over the telephone.

The final survey consisted of 24 questions which fell within four broad categories; 1) General management, 2) Feeding, 3) Biting and 4) Enrichment. These 24 questions were closed but as it was a telephone interview, all respondents were able to expand on the questions asked, providing additional information. At the end of the interview they were also asked if they had any additional comments. These quotes were all recorded and quantified by looking at the prevalence of certain themes or words. General management included questions regarding herd size, breeds used, ventilation type etc. The Feeding section included questions about the type of feed provided, the trough design, and cleaning of the water system. In the biting section respondents were asked to rank between 1 (not very serious) and 4 (very serious) what they considered to be the biggest negative consequence of tail and ear biting. They were also requested information regarding what factors they felt were important factors in the prevention of tail biting (1 = not at all important-7 = very important) and what they do in the event of a tail biting outbreak (1 = least often done 7 = most often done). Finally, the section on enrichment included questions regarding what type is used, the reasons why, and whether any other types would be considered. Respondents were asked to rank their biggest considerations when choosing enrichment from 1 (least influence) - 7 (biggest influence). (See Appendix for full list of questions).

Prior to the launch of the survey, pig producers were informed about it through the Teagasc Pig Departments Newsletter (August 2015) which is distributed by email to all producers who avail of the advisory service. The contact details of 70 producers were obtained from the Teagasc advisory service. These consisted of producers who had previously indicated that they were happy to take part in surveys for research purposes. All the producers (*n* = 70) were contacted by telephone and asked to participate in the survey which took a maximum of 10 min. The same researcher conducted all the surveys, with all being completed over the telephone.

### Statistical analysis

All statistical analyses were performed with IBM SPSS statistics 22. Descriptive data are given as median and interquartile range, and average and standard deviation. Where statistical analyses were carried out, non-parametric tests were used. Correlations between herd size, perceived seriousness of biting, acceptable levels of biting and frequency of biting on farms was tested using Spearman rank correlations. This was also used to investigate whether there was a correlation between tail and ear biting and outbreaks in the weaner and finishing stages. The difference in the mean score given to various preventive measures, as well as the difference in perceived importance of remedial measures was tested using Friedman’s two-way analyses followed by Wilcoxon rank sum test for pairwise comparisons. The analysis was also based on the methods used in Valros et al. [20] Finnish study to allow comparison.

## Results

### Management

A total of 70 producers were contacted, of which 58, from 21 Irish Counties participated in the survey. This represents approximately 16% of producers in Ireland. The 12 who didn’t participate, from seven counties, said that they were too busy to partake at that time. Since they did not take part and due to confidentiality regulations, no further information was available on these 12 producers. The surveyed farms ranged in size from units with 90 sows to those with 3000, in total representing 39,755 sows (approx. 26.7% of the sows in the country). Thirty-nine percent of those surveyed had < 500 sows, 46% 500–1000 and 15% > 1000 sows. The average herd size (710 ± 597 sows) was representative of the national average in 2015 (776 pigs; [38]). All farms surveyed were farrow to finish farms.

In 69% of these units, growing pigs were kept in mixed sex groups that were continually remixed at each stage (Table 1). Slatted systems predominated, with 4% of respondents having partly slatted pens and the remaining 96% having fully slatted pens. Of those that cleaned water dispensers (41%), this was carried out on average every four (± 9) weeks or at a batch level. Home milling was not common amongst respondents with 5% milling their own feed. Dry pelleted feed was most commonly fed to weaners (58%). In finisher pigs, pellets were used in 31.6% of cases and wet/dry feed 38.6% (Pig feeders for wet/dry feed have a drinker in the trough so the pig can mix dry feed with water themselves). Pigs were all fed ad libitum with 12.3% of respondents believing that all pigs could gain access to the feeder at the same time in the first stage, 8.8% in the second and 14% in the finishing stage. All respondents responded that they docked the tails of piglets with the majority (54%) removing 2/3 of the tail (range 50–90%). This was generally carried out at three days of age (range 0–5).

**Table 1:** Descriptive statistics of general management of 58 Irish farrow-to-finish farms participating in a survey about tail biting in their growing pigs

Question	Answer	Range
Most common maternal line	Landrace/Large white 60% Duroc 7% Hampshire 3% Unknown* 30%	
Most common meat line	Danbred 29% Duroc 17% Maxgro 17% Landrace/Large white 12% Hampshire 5% Unknown* 20%	
Weaning age (days)	28 (±1.8)	21–35
Average 1st stage (approximately 0–47 days from weaning) weaners per pen	42 (±25)	12–134
Average 2nd stage (approximately 47–81 days from weaning) weaners per pen	32 (±17)	12–100
Average finishers (approximately 81–159 days from weaning) per pen	26 (±11)	8–60
Percentage kept in mixed sex groups	69%	
Percentage kept in same group from weaning	34%	
Percentage mixed at all stages	66%	
Most common ventilation in first stage weaners	Natural 14% Mechanical 86%	
Most common ventilation in second stage weaners	Natural 13% Mechanical 87%	
Most common ventilation in finishers	Natural 23% Mechanical 77%	

*Unknown refers to cases where the breed was unknown by the producer. In some cases, the genetics company was referred to. When means are presented, they are (±Stdev)

There was no correlation between the herd size and either the frequency of biting (*p* = 0.20) (range-rare < 2 times to frequently > 5 times, mean: 1.46 ± 0.41, median: 1.33), levels considered acceptable (*p* = 0.38) (range 0-over 15%, mean: 1.36 ± 1.38, median: 1.5) or perceived seriousness of tail or ear biting (p = 0.38) (range 1 (not serious) - 7 (very serious), mean 2.88 ± 1.94, median: 2) (Spearmann Rank, *P* > 0.05). Tail biting and ear biting were ranked of equal concern (*p* < 0.01) with both given a median score of 2. There was a significant correlation between the frequency of tail biting that was occurring and the levels of tail biting that was considered acceptable by respondents; thus, the more tail biting observed, the higher the level that was considered acceptable (r = 0.340, *p* = .01).

### Biggest negatives of tail biting

All respondents commented at some stage during the interview, on the sporadic, unpredictable nature of outbreaks and the fact that there was no definite solution when it does occur. All factors were considered negative in relation to tail biting with all scoring an average greater than 3. However, condemnation and loss of productivity remained the biggest concerns with both receiving an average of 4 (most serious). Condemnation in particular was given a score of 4 (most serious) by 79% of respondents. Other negatives (21%) discussed included the pressure on already limited space caused by having to isolate pigs, the welfare of the bitten pigs and loosing pigs due to paralysis.

### When biting behaviour was observed to occur

Only two farms had experienced no tail or ear biting in the last year. There was no obvious difference between these farms, and the remainder of the farms surveyed, or similarities between the two (e.g. one fed all stages by liquid long trough, and one by dry pellets; one had approx. 100 pigs/pen, the other 15 pigs/pen at all stages). Of the farms that had experienced tail biting in the last year (51 of the 58 farms), 86% had also experienced ear biting. Of these incidences, farmers experienced the greatest amount of ear biting in the second stage and the highest incidences of tail biting in the finishing stage (Table 2).

**Table 2:** The percentage of the 58 Irish farrow-to-finish farms who participated in the survey that had experienced ear or tail biting amongst their growing pigs at each stage during the last year

	Ear biting (%)	Tail biting (%)
1st stage	53	26
2nd stage	72	63
Finishers	39	72

In 52% of cases, tail and ear biting wasn’t continuously occurring on farms and instead occurred sporadically, at certain times of the year or amongst certain batches. For instance, there was a significant correlation between outbreaks of tail biting in the 2nd stage and ear biting at this stage, and both ear and tail biting in the finishing stage. However, while ear biting in the finishing stage was correlated with outbreaks in the 1st stage, this was not true for tail biting (Table 3).

**Table 3:** Correlation between the stage’s that the 58 Irish farrow-to-finish farms participating in the survey, reported having outbreaks of tail and ear biting amongst the growing pigs on their farm

		1st stage		2nd stage		Finishers	
		**Tail**	**Ear**	**Tail**	**Ear**	**Tail**	**Ear**
1st stage	Tail		r = .396, *p* = .003	r = .292, *p* = .032	NS	NS	r = .318, *p* = .019
	Ear	r = .396, p = .003		NS	r = .436, *p* = .001	NS	NS
2nd stage	Tail	r = .292, p = .032	NS		r = .580, *p* = .000	r = .591, *p* = .000	r = .392, p = .003
	Ear	NS	r = .436, p = .001	r = .580, p = .000		r = .317, *p* = .020	NS
Finishers	Tail	NS	NS	r = .591, p = .000	r = .317, p = .020		r = .495, p = .000
	Ear	r = .318, p = .019	NS	r = .392, p = .003	NS	r = .495, p = .000	

**Fig. 1:**
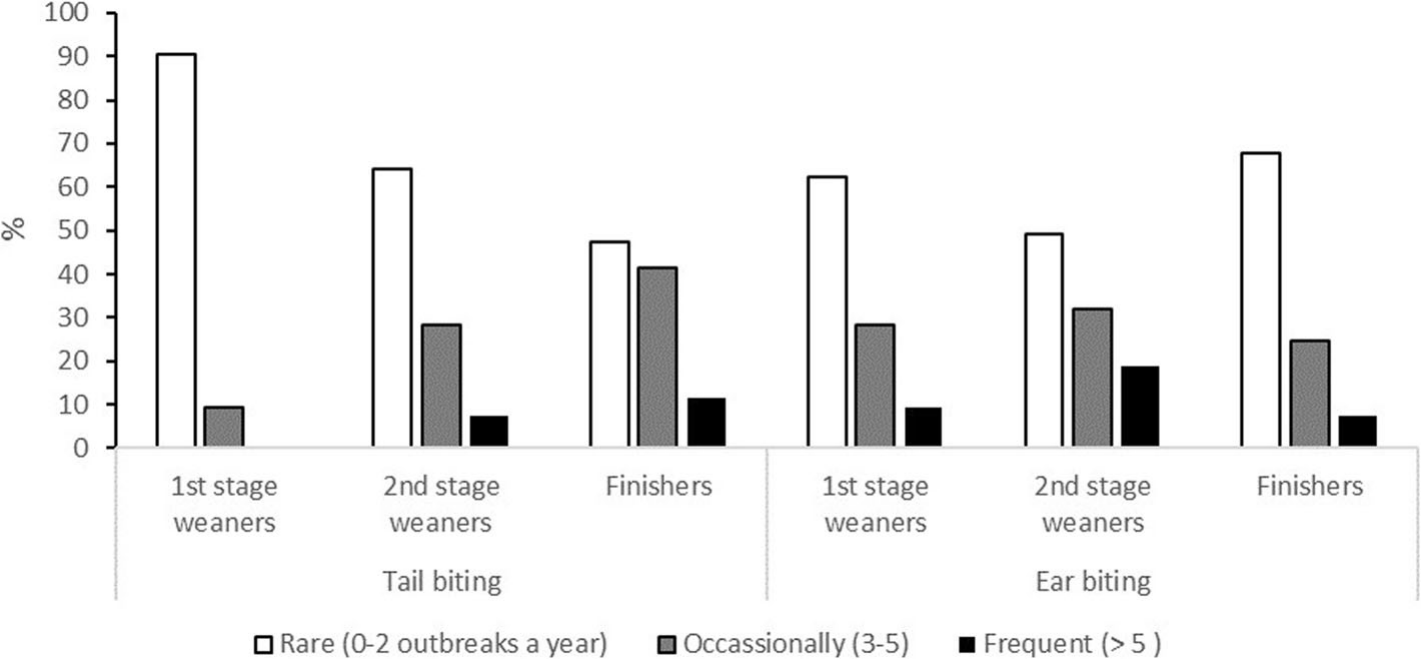
The frequency (%) at which 58 Irish farrow-to-finish farms participating in the survey about biting in their growing pigs, observed tail and ear biting at each stage of growth

Ear biting was reported to occur most often during the second stage, with few incidences of it occurring once the pigs had entered the finishing stage (Fig. 1). In contrast, tail biting, while still observed in the first and second stage, was reported by the majority of respondents to be most common in the finishing stage (Fig. 1).

### Tail biting prevention

Noise was a factor that none of the respondents had previously considered. As bedding type material is not compatible with the slatted systems used by respondents, none felt like they could comment on the benefit of straw as a bedding material, in reducing tail biting. However, when asked about bedding type material, all respondents felt that it probably would help if they could use it. Certainly, most of the respondents felt that tail biting was a symptom of another problem, with taking care of animal health, restricting animal density, maintaining an even quality of feed and content and managing air movement felt to be the most effective preventive measures (Table 4). There was no significant difference between the scores given for the eight categories ranked highest (taking care of animal health to water available to all pigs), but ‘taking care of animal health scored significantly higher (*P* = 0.001-Wilcoxon signed ranks test)) than the remaining categories (good quality pigs-managing noise level) (Table 4).

**Table 4:** Perceived importance to 58 Irish farrow-to-finish pig farms of the different preventive measures suggested in the survey to combat biting behaviour in growing pigs

Category	Mean ± standard deviation (average score of category)	Median (interquartile range*)
Taking care of animal health	6.35 ± 1.62^a^	7 (6)
Restricting animal density	6.26 ± 1.76	7 (1)
Even quality of feed	6.25 ± 1.83	7 (2)
Correct feed content	6.18 ± 1.87	7 (4)
Managing air movements (draughts, cold air pockets-e.g when slurry is low)	6.16 ± 1.86	7 (0)
Managing air quality	6.04 ± 1.69	7 (1)
Appropriate temperature in pen (not too hot or cold)	5.89 ± 2.00	7 (0)
Maintaining enough feeding space (enough space for each pig at the trough)	5.88 ± 1.96	7 (4)
Water available to all pigs	5.77 ± 2.04	7 (6)
Good quality pigs (healthy, evenly grown)	5.12 ± 2.35^b^	7 (2)
Use of objects for manipulation	4.74 ± 2.32^b^	5 (2)
Managing pen hygiene/cleanliness	4.39 ± 2.68^b^	5 (4)
Avoiding mixing of animals	4.00 ± 2.61^b^	3 (0)
Knowing the background of the piglet (housing and management in the farrowing unit)	3.56 ± 2.60^b^	3 (0)
Adequate light levels	3.28 ± 2.37^b^	3 (3)
Adjusting natural light from windows	3.26 ± 2.32^b^	3 (3)
Breed of pigs	3.02 ± 2.73^b^	2 (4)
Use of bedding type material	2.49 ± 2.48^b^	1 (6)
Feeding always at the same time	1.82 ± 2.72^b^	1 (0)
Managing noise level	1.70 ± 1.56^b^	1 (5)

Different letters (a, b) indicate *P* < 0.01 based on pair-wise comparisons

*Score 1-not important-7 very important

### Pig gender, season and breed

Eighty three percent of producers noted no difference in the likelihood of being a victim of biting between the sexes of the pigs. Of those that had (17%) 6% noted a higher incidence of female pigs biting and 11% in male pigs. The same was true in the case of season, with 53% observing no difference. However, some respondents (10%) reported noticing higher incidences when the seasons changed, or when there were extremes between day and night temperatures. In terms of breed, many producers noticed no impact (76%). Twenty-four percent highlighted problems in the past with Hampshires stating that they had been farming Hampshire’s but has switched to Duroc’s because of the levels of tail biting*.* However, others either didn’t comment on breed specific problems, or felt that despite having more biting with Hampshires the positives of the breed outweighed it.

### In the event of an outbreak

Respondents were asked to rank what they do in the event of an outbreak between 1 (wouldn’t do)-7 (would do). While 24% of the respondents expressed a wish to be able to remove the biter, identifying the biter was deemed difficult. They therefore concentrated on removing the bitten pig. This was ranked significantly higher than all other measures (*p* < 0.05-for all comparisons) (Table 5). The second most common course of action was to add additional objects to distract and occupy the pigs. This was ranked significantly higher than all other categories (p < 0.05-for all comparisons).

**Table 5:** What 58 Irish farrow-to-finish farms participating in the survey do on a pen level in the event of a tail biting outbreak, ranked from 1 (wouldn’t do) to 7 (would do)

Measure	Mean (± Sdev)	Median (Interquartile range)
Remove bitten (4)	6.39 ± 1.35 ^a^	7 (0)
Add object (5)	5.63 ± 2.22 ^b^	7 (2)
Remove biter (2)	4.40 ± 2.43 ^c^	3 (4)
Identify biter (1)	4.40 ± 2.37 ^c^	5 (4)
Anti-biting substance (7)	3.70 ± 2.60 ^c^	5 (5)
Reduce density (6)	3.49 ± 2.38 ^c^	3 (6)
Add bedding (3)	2.09 ± 2.06 ^c^	1 (1)

Numbers in parentheses next to the measure indicate the ranking for each measure by Finnish farmers [20]. Different letters (a, b, c) indicate *P* < 0.01 based on pair-wise comparisons

**Fig. 2:**
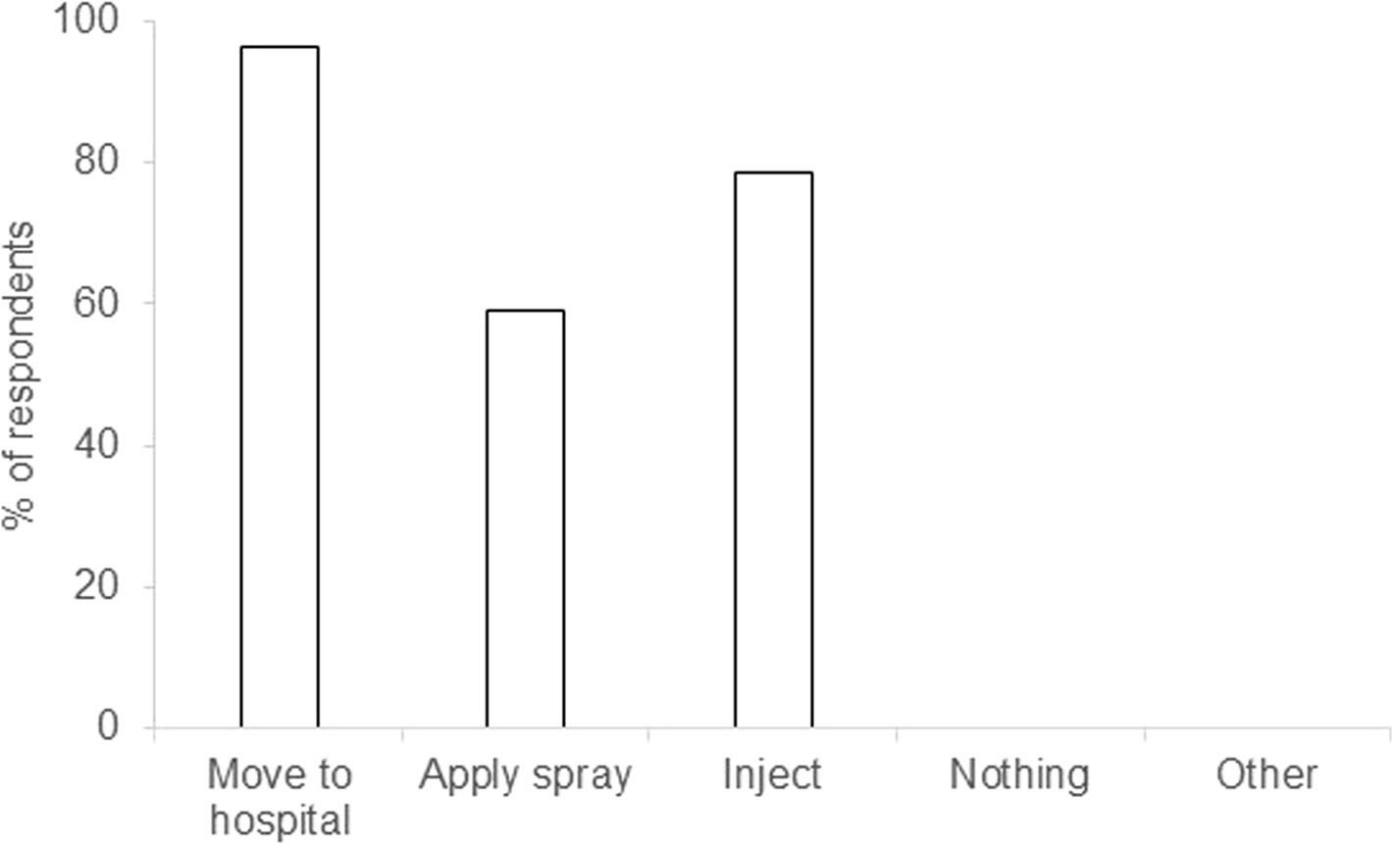
The course of action taken by 58 Irish farrow-to-finish farms participating in the survey about biting in their growing pigs, took towards bitten pigs. The y-axis displays the percentage ofrespondents that utilised each method

Respondents were asked yes or no, in relation to common measures for treating bitten pigs (Fig. 2). In most cases bitten pigs would be placed in the hospital pen until the tail area dried up. In some cases, the bitten pig would also be injected with antibiotics (Fig. 2). Fifty seven percent of respondents found sprays useful, the remainder (43%) did not find them effective.

### Enrichment currently used

**Fig. 3:**
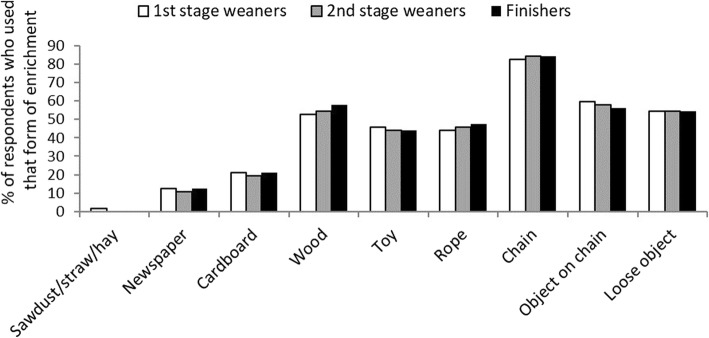
The bedding type material and manipulable objects currently used by 58 Irish farrow-to-finish farms to prevent tail biting in their growing pigs. The y-axis displays the percentage of respondents participating in the survey about biting, that utilised each method

Twenty-one percent of respondents believed that boredom has a big effect on tail biting and have found various forms of enrichment successful in preventing tail biting from occurring (Fig. 3). Only one respondent didn’t use any enrichment, with 86% using more than one type. Chains were the most common (83%), which alone are not considered sufficient forms of enrichment under EU law as they are not destructible. Those who didn’t use chains, utilised wood, commercial toys or rope instead. Sixty-five percent of respondents added additional enrichment such as wood following an outbreak of biting. While chains were used most commonly because of their durability and ease of use, wood was also highlighted as successful enrichment by 65% of respondents.

### Considerations when selecting enrichment

**Fig. 4:**
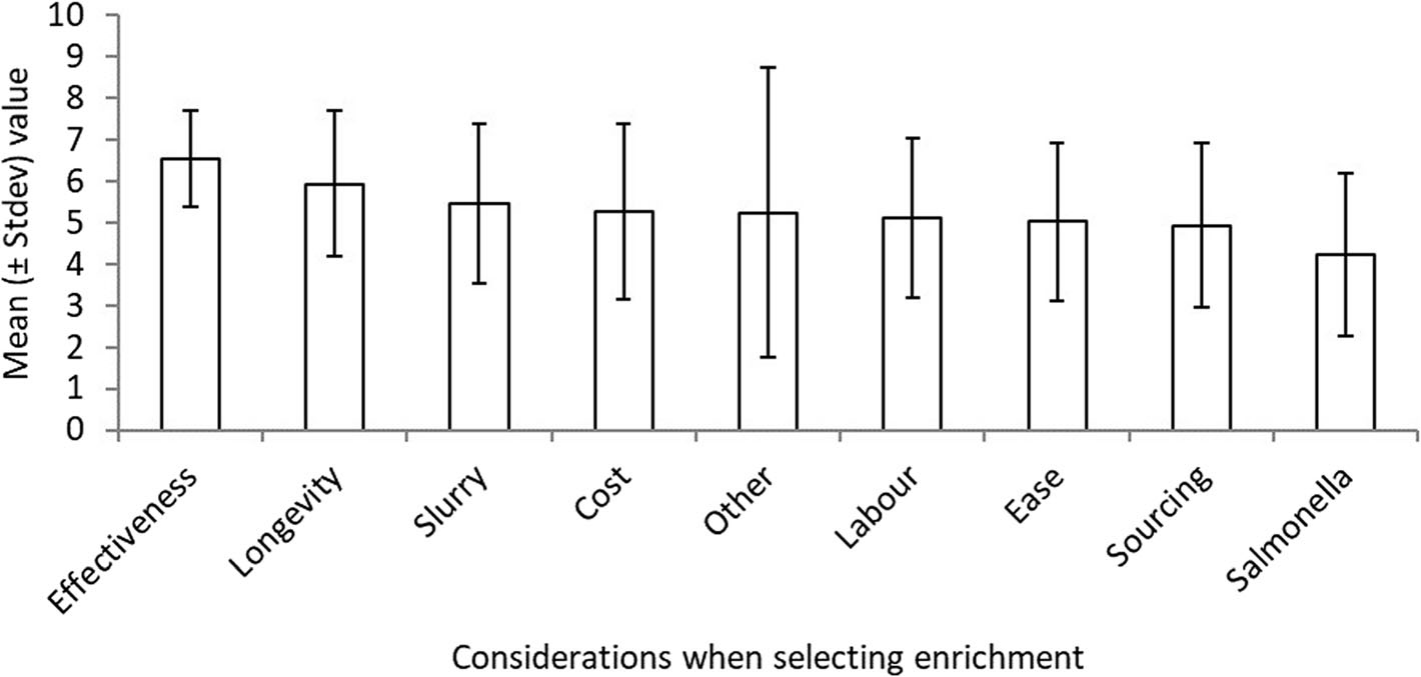
The biggest considerations of 58 Irish farrow-to-finish farms participating in the survey about biting in their growing pigs when selecting bedding type material and manipulable objects. The y-axis displays the average score (±stdev) of importance given by respondents to the given factors

The main considerations when choosing enrichment were that it was effective (83%) and lasted with both receiving a mean score of 6 (1–7 with 7 being most important). (Fig. 4). There was a preference amongst respondents for enrichment that could be constructed from existing materials found around the farm. Durability was also a common reason flagged for wood being a preferred form of enrichment. Longevity of the enrichment was considered an important consideration for 97% of respondents.

Eighty one percent of respondents would consider using additional enrichment to what they are currently using; the additional materials they would consider were similar to the existing enrichment that was being used. Thirty-six percent of producers preferred hanging items, mainly due to concerns that the object would get dirty and stop being desirable to the pigs if placed on the floor. Seven percent of respondents were also concerned about salmonella risk with ground objects and the objects getting in the feeder and blocking the probe.

## Discussion

In the current study, only two farms had experienced no tail or ear biting in the last year highlighting the widespread occurrence of the problem in Ireland. This was despite the fact that tails were docked in all farms included in the study, which should reduce the risk of being bitten [39]. These data confirm previous results from Irish factory studies that a high proportion (60–70%) of docked pigs have detectable tail lesions, and thus that tail docking appears to be ineffective in eliminating tail biting [1, 2]. This supports the argument that tail docking is simply masking the real underlying problems in housing and management that are leading to tail biting [8, 40].

It has been hypothesised that when tails are docked, damaging behaviour is redirected to other areas like the ears [41]. This appears to be a legitimate concern as in the current study ear biting was common, and all tails were docked. Prevalence of tail and ear biting in the 1st and 2nd stage were significantly correlated, and tail biting in the 2nd stage was significantly correlated with tail and ear biting in the finisher stage, confirming that there may be a link between the two [9].

Given the high economic costs of biting (€1.69/pig; [2]), it is not surprising that for 79% of respondents, condemnation was the biggest negative associated with tail biting, closely followed by loss of productivity. The importance of catching biting at an early stage was also highlighted. Indeed, the benefits of identifying and attempting to stop tail biting outbreaks quickly has been scientifically evaluated by Chou et al. [42]; that study found that the chances of an intervention strategy being successful was more related to the proportion of biters/victims in the pen, than the type of intervention (removing the biter, or the victim, or adding additional enrichment) used. The difficulty of detecting whether a pig would be condemned was also highlighted by respondents, as damage that happens early in the production cycle could looks visually healed by slaughter. Tail lesions were significantly associated with carcass condemnation in an Irish study, where abscessation accounted for up to 70% of total carcass condemnations [2]. Moreover, Camerlink et al. [43] reported that pigs that received oral manipulation (such as tail biting) grew less well irrespective of the severity of wounds, which corresponded to a weight difference of approximately 4 kg at the end of the finishing period.

In the current study, tail biting was reported to occur more often as pigs aged, peaking in the finishing stage. Ear biting in contrast peaked in the 2nd stage. This self-reporting by producers is consistent with previous research in Ireland carried on 31 commercial farms [44], and thus it appears that producer perception is likely to be accurate.

### Biting prevention

Unlike the results from the Finnish survey [20], Irish producers did not recognise the importance of many of the preventive measures presented to them; indeed 8 of the measures scored lower than the median (score of 4). This is likely due to the differences in pig production systems in the two countries; Finnish farmers rear pigs without docking, and thus are likely to be more aware of preventive measures which have been identified scientifically, and which they may experience themselves, than the cohort of Irish farmers which took part in this survey. Nevertheless, what was similar between the two surveys, and with a survey carried out with Dutch farmers [24] was the rank order of the prevention measures; producers from all three countries ranked taking care of animal health, and managing air movement as being highly important. The link between biting and pig health is as yet poorly understood, but it is thought that sick animals are unwell and/or feel stressed and seek an outlet to express this negative feeling which may lead them to increase manipulatory behaviour of their pen mates. Poor health could also cause pigs to respond less to general manipulation by their pen mates thereby placing them at higher risk of injury. There is also limited, though growing, evidence that the role sickness plays in damaging behaviour is mediated via cytokines produced as part of the inflammatory response [45]. Moreover, associations between tail lesions and other disease associated lesions such as pleuritic lesions, lesions of enzootic pneumonia and external carcass abscesses have been observed at the factory, providing further evidence that a link exists [46, 47].

These opinions diverge from the general scientific consensus; in fact, in the 2007 EFSA scientific opinion on tail biting, access to manipulable objects was ranked most highly in importance. Similarly, in a survey of producers carried out in the UK in 2007 [19], this was ranked highly with 86.6% of producers identified ‘boredom’, 78.9% lack of bedding type materials, and 71% ‘bad mood of the pig’ as either important or very important in causing tail biting. Our survey however, as well as the Finnish and Dutch one, did not include specific questions regarding the mental state of the pig.

Restricting animal density has been identified as having a significant influence on the prevention of tail biting by EFSA [23]. In a UK survey 76.5% of producers considered a high stocking density to be a risk factor for tail biting [19]. Likewise, producers in this survey ranked it as the second highest risk factor, the same position as Dutch [24], and Swedish pig producers [48]. In countries where docking is completely prohibited (e.g. Finland, Sweden, Norway, Switzerland) maximum stocking densities for commercial pigs are lower than the EU standard; this could perhaps be one of the reasons why Finnish farmers ranked stocking density lower, the 11th most important out of 20 risk factors. Maintaining a low stocking rate is complicated by the genetic selection for increased output per sow per year which has occurred in recent years; for instance, the number of piglets born alive per litter has increased from 10.8 in 2000 to 13.5 in 2017 in Ireland (information sourced from the Teagasc PigSys recording system). Thus, to ensure that stocking density limits are not breached, and if producers aim to mitigate risk of biting by decreasing stocking density, it may be necessary to either reduce sow numbers, or construct additional facilities.

Following taking care of animal health and restricting animal density, the next two prevention measures (3rd and 4th) identified by producers were feed quality and type. Maintaining enough feeder space was in the top half of the list, at 8th. Just 5% of respondents were home milling, and thus control over feed quality is somewhat limited, which could have contributed to concerns about quality. However, Finnish and Swedish producers also ranked feeding issues highly. In the Finnish survey feeding space was considered most important, feed quantity 5th, and feed quality 7th [20], and in the Swedish survey feed composition/equipment was the most commonly reported cause of biting in finishing pigs [48]. In contrast Dutch producers [24] ranked feed and feeding system least important out of a list of 10 risk factors, and producers in the UK also ranked problems with the feed supply 11th out of 14 risk factors. In Finland the long trough system is predominant, and it is possible that this is partly the case because producers recognise the importance of reducing competition at feeding. Sudden tail-biting is commonly seen when pigs are unable to access a desired resource, such as food [49] and social conflicts around the feeder can change feed intake related behaviour of pigs, such as the number of feeder visits, duration of the visits and feeding rate [50]. Moinard et al. [16] showed that five or more pigs per feed space increased the risk of tail biting by a factor of 2.7. Similarly, Palander et al. [18], observed that 60% of tail biting, was conducted by pigs which had limited feeder access.

Draughts, poor ventilation and bad air quality ranked 5 to 7 in the list of tail-biting risks. The survey of Dutch farmers reported that a stable climate was considered to be the most important risk factor for tail biting [24], and it ranked third amongst Finnish farmers [20]. Experimentally Scheepens et al. [51] found a five times higher level of redirected exploration behaviour towards pen mates in the event of an unexpected occurrence of draught, which supports these farmer assessments. Thus, adequate control over the environment seems to be essential in reducing risk.

### General enrichment use

Most respondents stated that the effectiveness (83%) and longevity (97%) of enrichment were their biggest considerations. This is not surprising when considering that some units had up to 3000 sows; thus, many preferred homemade devices or items already on the farm. The use of ready available materials was also apparent in the survey of Finnish producers [20] where respondents mentioned materials such as used car tyres, pieces of watering hose, stones, empty plastic containers, and used boots and shoes. In contrast, 99% of Swedish producers reported using straw, and the remaining 1% used wood shavings or sawdust instead [48]. In that survey, the observance of outbreaks was much lower than in the Irish one despite the pig’s tails remaining undocked, with only 50% of nurseries reporting bitten pigs, and 88% of finishing units, which may be linked to the provision of more appropriate enrichment.

Many producers felt that wood met the criterion of being durable, while yet stimulating on-going use by the pigs. Telkanranta et al. [52] concluded that horizontally suspended pieces of fresh wood can increase object exploration and reduce tail and ear biting in commercial pig farming. Nevertheless, even within the broad category of ‘wood’ there can be differences in rate of use, and in the amount of object exploration that it stimulates. Chou et al. [53] found that softer wood types are more engaging for pigs yet wear away more quickly.

### Strategies to deal with outbreaks

Many respondents, despite preferring to remove the biter, removed the bitten pig. Correspondingly, in a British survey 67% of producers reported that they remove the bitten pigs from the pen while only 43% said they removed the biter [54]. In contrast, most of the Finnish, Swedish and Dutch farmers identify and/or remove the tail biter. Recent work by our group compared three tail biting control protocols; removing the victim, removing the biter, or addition of manipulable objects (ropes). We found that providing additional manipulable objects reduced the duration of outbreaks more than the other protocols, but that all three were equally effective in stopping them [42]. The advantage of adding enrichment materials is that fewer hospital pens are needed for removed pigs, and indeed 81% would consider adding additional enrichment if it proved to be effective and viable. Unfortunately, many respondents felt that the use of bedding type materials such as straw was not possible due to having a fully slatted system. This accentuates the importance of considering the limitations of some forms of enrichment and the need to consider what producers are willing to/able to use.

## Overall conclusion

Irish pig farmers reported high incidence of both tail and ear biting in their docked pigs, highlighting the extent of the problem, the link between ear and tail biting and the fact that tail docking may merely mask other systematic management problems. Of significance, is that although Irish farmers consider tail biting a serious problem, they appear to be less able to identify preventive measures for tail biting than producers from Finland and The Netherlands. Nevertheless, they did rank measures in broadly the same order as producers from the different countries and recognised that high stocking densities and issues relating to food type and provision system are significant. Thus, simply improving awareness of preventive measures amongst producers could significantly help the industry comply with EU legislation pertaining to tail docking and enrichment provision for pigs.
